# Ultra-Processed Food Is Positively Associated With Depressive Symptoms Among United States Adults

**DOI:** 10.3389/fnut.2020.600449

**Published:** 2020-12-15

**Authors:** Liwen Zheng, Jing Sun, Xiaohui Yu, Dongfeng Zhang

**Affiliations:** Department of Epidemiology and Health Statistics, School of Public Health, Qingdao University, Qingdao, China

**Keywords:** depressive symptoms, ultra-processed food, dose-response, cross-sectional study, NHANES

## Abstract

Ultra-processed foods (UPFs) are popular in the United States. In recent years, there has been an increasing interest in the health impact of UPF. This study is conducted to assess the association between UPF consumption and depressive symptoms among United States adults. Data were collected from the National Health and Nutrition Examination Survey 2011–2016. Dietary data were obtained through 24-h dietary recall interviews. Depressive symptoms were detected by a nine-item Patient Health Questionnaire; participants with more than 10 points were diagnosed with depressive symptoms. Results of logistic regression revealed a positive association between UPF consumption and depressive symptoms. The study suggests that UPF may increase the risk of depressive symptoms, particularly in people with less exercise.

## Introduction

Food processing aims to improve food availability, safety, digestibility, transportability, and storage life (1). Since the mid-nineteenth century, the mechanization of the food industry has made it possible to produce, transport, and sell processed foods on a large scale. To better understand the impact of the nature, purpose, and extent of food processing on human health and disease, a novel food classification method—NOVA (a name, not an acronym) was proposed. An updated version of NOVA classified all foods into four groups (2): (1) unprocessed or minimally processed foods; (2) processed culinary ingredients; (3) processed foods; (4) ultra-processed foods (UPFs) and drink products. Among them, UPFs are attracting increasing attention.

UPFs are essentially industrial formulations mostly or entirely made from industrial ingredients, with little or no whole foods. They often contain substances not used in home cooking, especially the additives for sensory properties of food (3). Typical UPFs include carbonated beverages, bagged snacks, mass-produced packaged bread and buns, and ice cream. Because of their super-palatability, convenience, and long storage life, UPFs dominate the food supply in high-income countries, particularly in the United States (US), where UPFs account for 57.5% of total energy intake (4). At the same time, UPF consumption is increasing rapidly in middle-income countries (5).

UPF producers prioritize taste, cost, storage, and stability during transport, whereas neglecting nutritional quality (6). UPFs are common in the western dietary pattern and generally rich in total fat, saturated fat, added sugar, and salt, whereas poor in fiber and vitamin density (7, 8), which is detrimental to mental health (9, 10). Beyond poor nutritional quality, UPFs also contain all kinds of additives, along with neo-formed contaminants produced during food processing and packaging (11–13), some of which may have an adverse effect on intestinal flora (14, 15), inducing the development of inflammation-associated diseases (16, 17), such as depression.

In recent years, the impact of high UPF consumption has aroused widespread public concerns, stimulating extensive researches to investigate adverse health outcomes related to UPF. Researches have demonstrated an association between UPF consumption and increased risk of all-cause mortality (18–20), cancer (21), type 2 diabetes (22), and cardiovascular diseases (23). Additionally, positive associations with frailty (24), overweight/obesity (25) were reported in other studies. Among these studies, two European studies explored the association between UPF and mental disorders (26, 27). However, both two studies were conducted in a population with relatively low UPF consumption. There is a lack of a large-scale study to assess the association between UPF consumption and depressive symptoms in the US population. Thus, we conducted this study to evaluate the relationship between UPF consumption and depressive symptoms in US adults aged more than 20 years.

## Materials and Methods

### Data Source, Population, and Sampling

This study used data collected from the National Health and Nutrition Examination Survey (NHANES), which is administered by the National Centers for Health Statics at the Centers for Disease Control and Prevention. NHANES is conducted to assess the health and nutritional status of the US population of all ages. Data are collected using a complex, multistage probability sampling design to make the sample nationally representative. Participants received a detailed interview in their home and physical examination, dietary survey, and clinical laboratory tests at a mobile examination center on another day. All participants provided written informed consent, and the Research Ethics Review Board approved the study protocol.

**Figure 1:**
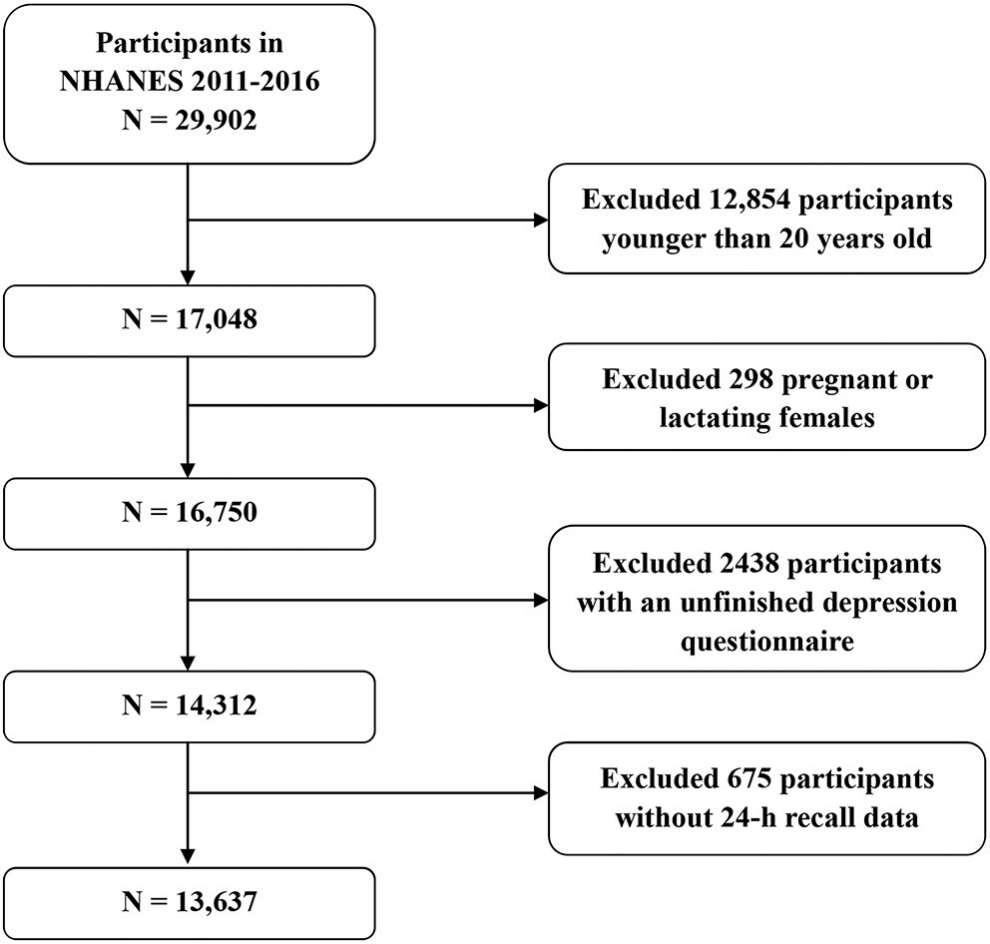
Flow chart of screening process for the selection of the study participants.

Data from three survey cycles (2011–2012, 2013–2014, and 2015–2016) were analyzed in this study. A total of 29,902 respondents participated in three survey cycles. The response rates of data collected through interviews in the three survey cycles were 72.6, 71.0, and 61.3%, respectively. In this study, we excluded 12,854 participants younger than 20 years old, 298 pregnant or lactating females, 2,438 participants with an unfinished depression questionnaire, and 675 participants without 24-h recall data. Finally, 13,637 individuals were included in our study (Figure 1).

### Dietary Assessment

Interviewer-administered 24-h dietary recall interview was used to estimate the consumption of foods and beverages. The validity of 24-h dietary recall has been proved in biomarker-based studies (28, 29). All participants are eligible for an in-person dietary interview by trained interviewers in the mobile exam center. The effective Automated Multiple-Pass Method was used to collect dietary data (30). The Automated Multiple-Pass Method is computerized with a five-step multiple-pass approach: collect a quick recall list of foods consumed the previous day, probe for forgotten foods, collect time and occasion of eating, collect detailed information of consumed foods, and final probe. Daily intakes of nutrients and energy were calculated based on self-reported consumed foods, according to the guidance of the Food and Nutrient Database for Dietary Studies.

Twenty-four-hour dietary recall is not representative of usual dietary habits. For reflecting participants' diet more precisely, a sensitivity analysis was conducted (Supplementary Table 2). During the dietary survey, all participants were asked, “was the amount of food that you ate yesterday much more than usual, usual, or much less than usual;” only participants who answered “usual” were included in the sensitivity analysis.

### Food Classification According to NOVA

NOVA classifies all foods into four categories according to the degree of processing (2). In this study, we mainly follow UPF with interest. UPFs are manufactured industrial foods, which usually contain abundant fat, saturated fat, sugar, and salt. Generally, UPFs do not contain or only contain a small percentage of unprocessed or minimally processed foods. According to the NOVA food classification system, we classified all food items as UPF or non-UPF. To ensure the accuracy of food classification and the consistency with other studies, we referred to the published literature (31, 32). The details of food classification are shown in the Supplementary Material.

The proportion of UPF in total energy intake (%UPF) was calculated to reflect participants' UPF consumption. UPF consumption was divided into quartiles as the exposure variable.

### Assessment of Depressive Symptoms

The nine-item Patient Health Questionnaire (PHQ-9) was used to detect depressive symptoms in NHANES. PHQ-9 consists of nine items, all based on the description of depression in the Diagnostic and Statistical Manual of Mental Disorders, fourth edition. Each item has four options: “No” (0 points), “several days” (1 point), “more than half of the time” (2 points), and “almost every day” (3 points). The total scores are the sum of the scores of all the items, ranging from 0 to 27. In the present study, participants whose PHQ-9 score ≥ 10 were classified as depressive symptoms. This criterion has been confirmed to have good specificity and sensitivity (33). Additionally, in sensitive analysis, individuals who self-reported using antidepressants have also seemed depressive symptoms for testing the stability of the results (Supplementary Table 3).

### Covariates

For controlling the potential confounding factors, we adjusted some covariates in multivariate models. Sociodemographic characteristics included sex, age (20–44 years, 45–59 years, 60 years, or older), race (Hispanic, non-Hispanic white, non-Hispanic black, non-Hispanic Asian, or other races), educational level (below high school, high school, or over high school), annual family income (<$20,000, $20,000–<$45,000, $45,000–<$75,000, ≥$75,000), and marital status (married/living with a partner, divorced/separated/widowed/single). Body mass index (BMI) was calculated as weight (kilogram) divided by height squared (square meter) and categorized as underweight or normal weight (<25 kg/m^2^), pre-obesity (25–<30 kg/m^2^), or obesity (≥30 kg/m^2^).

Lifestyle characteristics were also considered. Physical activities were evaluated by the Global Physical Activity Questionnaire. Activity levels were divided into active (more than 300 min of moderate-intensity physical activity a week), moderately active (150–300 min of moderate-intensity, or 75–150 min of vigorous-intensity aerobic physical activity per week), and active (<150 min of moderate-intensity physical activity a week). With regard to smoking status, participants were categorized as current smoker, former smoker, and never smoker. Drinking alcohol was defined if they had at least 12 alcohol drinks a year.

In addition, we adjusted some chronic diseases. Blood pressure was measured in the mobile exam center and calculated by the mean of three blood pressure measurements; hypertension was defined as systolic pressure ≥ 130 mmHg and diastolic pressure ≥ 80 mmHg. About two-thirds of NHANES participants did not finish fasting blood glucose measures, so the definition of diabetes was based on self-reported clinical diagnosis. Heart disease and chronic bronchitis were also self-reported.

### Statistical Analysis

According to the official guidance of NHANES, we constructed new simple weights by taking one-third of the 2-year weights. New weights were used in an analysis to make an estimate representative of the US civilian non-institutionalized resident population.

We used weighted percentages or means for describing categorical and continuous variables, respectively. For comparing the distribution of sociodemographic characteristics, lifestyle, and dietary intake between the depressive symptoms group and non-depressive symptoms group, we used Cochran–Mantel–Haenszel chi-square test for categorical variables and Student's *t*-test for continuous variables. The multiple adjusted logistic regression model was used to calculate the odds ratios (ORs) and 95% confidence intervals (95% CIs) for depressive symptoms according to UPF consumption, with the lowest quartile as reference. Model 1 was adjusted for age and sex. Model 2 was adjusted for age, sex, race, educational level, annual family income, marital status, BMI, physical activity, smoking, drinking, hypertension, diabetes, heart disease, and chronic bronchitis. The significance of the linear trend was calculated using the median value of each quartile as a continuous variable in each model. In addition, we conducted stratified analyses to test differed associations among people with different physical activity levels. We also assessed the dose–response relationship by restricted cubic spline with knots at the 5th, 25th, 50th, 75th, and 95th percentiles of the exposure distribution, adjusted for all covariates. Stata 15.0 was used for organizing the data and statistical analyses. All reported probabilities (*p*-values) were two-sides with a statistical significance level of 0.05.

## Results

Table 1 described the demographic and behavioral characteristics of the 13,637 participants included in this analysis. In the analytic sample, participants consumed an average of 1,201 kcal/day of UPF consumption, equivalent to 55% of total energy intake. Depressed individuals tended to consume more UPF. Among all included participants, 1,208 (8.9%) of them met the definition of depressive symptoms. Women had a significantly higher prevalence (11.3%) of depressive symptoms than men (6.4%). Compared with individuals without depressive symptoms, those with depressive symptoms (PHQ-9 score ≥ 10) were middle-aged, less education, lower-income, more obese, and living alone. Depressed individuals were also physically inactive, and they were more likely to smoke. In addition, elevated UPF consumption was associated with low dietary quality (Table 2). People with high UPF consumption tended to intake fewer vitamins and trace elements (n-3 fatty acid, dietary fiber, vitamin C, vitamin E, folate, calcium, and zinc) but more saturated fats, sugars, and energy.

**Table 1:** Descriptive characteristics of the study participants, NHANES 2011–2016 (*N* = 13,637).

	**All participants**	**Without depressive symptoms: PHQ < 10**	**With depressive symptoms: PHQ ≥ 10**	***p-*value**
	**(*n* = 13,637)**	**(*n* = 12,429)**	**(*n* = 1,208)**	
Age (%)^a^				0.002
20–44 years	43.0 (40.8, 45.2)	43.1 (40.8, 45.4)	41.7 (37.1, 46.5)	
45–59 years	29.2 (27.7, 30.6)	28.6 (27.2, 30.0)	35.7 (31.1, 40.6)	
60 years and older	27.8 (26.3, 29.5)	28.3 (26.6, 30.1)	22.6 (19.4, 26.2)	
Sex (%)^a^				<0.001
Men	49.9 (48.9, 51.0)	51.0 (50.0, 52.0)	38.1 (33.5, 42.4)	
Women	50.1 (49.0, 51.1)	49.0 (48.0, 50.0)	61.9 (57.6, 66.5)	
Race (%)^a^				<0.001
Hispanic	14.3 (11.7, 17.5)	14.1 (11.4, 17.2)	16.9 (13.2, 21.2)	
Non-Hispanic White	66.6 (62.3, 70.6)	66.8 (62.5, 70.9)	63.8 (57.7, 69.4)	
Non-Hispanic Black	11.0 (8.9, 13.5)	10.9 (8.8, 13.4)	11.9 (9.0, 15.6)	
Non-Hispanic Asian	5.1 (4.0, 6.4)	5.4 (4.3, 6.7)	1.8 (1.2, 2.8)	
Other races	3.0 (2.6, 3.6)	2.8 (2.3, 3.4)	5.6 (3.8, 8.1)	
BMI (%)^a^				<0.001
<25 kg/m^2^	29.1 (27.1, 30.8)	29.4 (27.6,31.3)	25.3 (20.9, 30.6)	
25–<30 kg/m^2^	33.1 (31.7, 34.5)	33.8 (32.3, 35.3)	24.9 (20.7, 29.6)	
≥30 kg/m^2^	37.8 (36.1, 39.5)	36.7 (35.0, 38.5)	49.8 (44.9, 54.7)	
Marital status (%)^a^				<0.001
Married/living with partner	61.3 (58.9, 63.6)	62.9 (60.5, 65.1)	44.0 (40.0, 48.2)	
Widowed/divorced/ Separated/never married	38.7 (38.7, 41.1)	37.1 (34.9, 39.5)	56.0 (51.8, 60.0)	
Educational level (%)^a^				<0.001
<high school	14.3 (12.6, 16.3)	13.5 (11.8, 15.5)	23.3 (19.5, 27.5)	
High school	21.3 (19.7, 23.0)	20.9 (19.2, 22.6)	25.5 (22.2, 29.2)	
>high school	64.4 (61.4, 67.3)	65.6 (65.6, 68.5)	51.2 (46.6, 55.8)	
Annual family income (%)^a^				<0.001
<$20,000	16.4 (16.5, 20.6)	16.7 (14.9, 18.6)	37.6 (32.3, 43.2)	
$20,000–<$45,000	27.8 (26.2, 29.5)	27.3 (25.8, 29.1)	32.4 (28.1, 37.1)	
$45,000–<$75,000	20.5 (18.9, 22.2)	20.8 (19.1, 22.6)	17.6 (13.7, 22.1)	
≥$75,000	33.3 (30.3, 36.3)	35.2 (32.2, 38.2)	12.4 (9.1, 16.7)	
Physical activity (%)^a^				<0.001
Active	18.0 (18.8, 19.1)	18.4 (17.2, 19.6)	13.0 (10.5, 15.8)	
Moderate active	15.2 (14.1, 16.3)	15.7 (14.6, 16.9)	8.8 (6.9, 11.2)	
Inactive	66.8 (65.3, 68.4)	65.9 (64.2, 67.4)	78.2 (74.8, 81.2)	
Smoking status (%)^a^				<0.001
Current smoker	25.3 (23.8, 26.9)	25.6 (24.1, 27.2)	21.8 (18.2, 26.0)	
Former smoker	19.6 (18.4, 20.9)	17.7 (16.6, 18.8)	40.5 (35.7, 45.6)	
Never smoker	55.1 (53.5, 56.7)	56.7 (55.1, 58.3)	37.7 (33.0, 42.4)	
Had at least 12 alcohol drinks a year (%)^a^	78.3 (76.1, 80.0)	78.0 (76.0, 80.0)	79.2 (76.0, 82.1)	0.56
Current hypertension (%)^a^	36.1 (34.8, 37.4)	36.1 (34.7, 37.5)	35.8 (31.7, 40.1)	0.22
Ever had diabetes (%)^a^	10.5 (9.8, 11.4)	10.0 (9.2, 10.9)	16.5 (13.8, 19.5)	<0.001
Ever had heart disease (%)^b^	7.0 (6.4, 7.6)	6.5 (5.9, 7.1)	13.0 (10.6, 15.9)	<0.001
Ever had chronic bronchitis (%)^b^	6.1 (5.3, 6.9)	5.3 (4.7, 6.0)	14.4 (11.5, 17.9)	<0.001
UPF% (% of total energy intake)^b^	54.9 (54.0, 55.7)	54.5 (53.8, 55.3)	58.3 (56.0, 60.6)	<0.001

*All percentages and means are weighted; percentages include missing data*.

a *Categorical variables are represented as % (95%CI)*.

b *Continuous variables are represented as means (standard errors)*.

**Table 2:** Nutrient intake according to quartiles of UPF consumption among US adults aged 20 years, NHANES 2011–2016 (*N* = 13,637).

	**Ultra-processed food consumption (% of total energy intake)**				
	**Quartile 1**	**Quartile 2**	**Quartile 3**	**Quartile 4**	
	**(*n* = 3,392)**	**(*n* = 3,532)**	**(*n* = 3,317)**	**(*n* = 3,396)**	***P^c^***
Energy (kcal/day)	2,021 ± 944	2,157 ± 933	2,199 ± 903	2,210 ± 956	<0.001
Protein %^a^	18.0 ± 6.4	16.3 ± 5.3	15.3 ± 5.0	14.0 ± 4.4	<0.001
Total carbohydrates %^a^	44.4 ± 12.6	47.7 ± 10.7	48.8 ± 10.6	50.0 ± 10.4	<0.001
Total fats %^a^	33.6 ± 10.8	34.0 ± 8.9	34.3 ± 8.5	35.5 ± 8.4	<0.001
Sugars (g/day, 1,000 kcal)^b^	43.7 ± 24.8	51.7 ± 23.8	55.3 ± 24.4	57.2 ± 27.1	<0.001
Saturated fats (g/day, 1,000 kcal)^b^	11.6 ± 4.9	12.0 ± 4.3	12.5 ± 4.2	13.1 ± 4.2	<0.001
n-3 fatty acid (g/day, 1,000 kcal)^b^	1.00 ± 0.70	0.92 ± 0.55	0.87 ± 0.47	0.85 ± 0.43	<0.001
n-6 fatty acid (g/day, 1,000 kcal)^b^	7.9 ± 4.1	8.0 ± 3.3	8.0 ± 3.4	8.2 ± 3.4	0.01
Fiber (g/day, 1,000 kcal)^b^	9.8 ± 5.5	9.0 ± 4.4	8.1 ± 4.0	7.0 ± 3.4	<0.001
Vitamin A (μg/day, 1,000 kcal)^b^	364 ± 490	353 ± 354	319 ± 499	250 ± 241	<0.001
Vitamin C (mg/day, 1,000 kcal)^b^	51.1 ± 57.4	43.8 ± 43.7	39.0 ± 43.2	29.8 ± 37.8	<0.001
Vitamin D (μg/day, 1,000 kcal)^b^	2.7 ± 3.6	2.5 ± 2.7	2.3 ± 2.4	1.7 ± 2.0	<0.001
Vitamin E (mg/day, 1,000 kcal)^b^	4.8 ± 3.2	4.6 ± 2.9	4.2 ± 2.6	3.9 ± 2.8	<0.001
Folate (μg/day, 1,000 kcal)^b^	263 ± 150	266 ± 150	256 ± 165	246 ± 173	<0.001
Calcium (mg/day, 1,000 kcal)^b^	473 ± 239	479 ± 236	465 ± 220	444 ± 211	<0.001
Phosphorus (mg/day, 1,000 kcal)^b^	715 ± 195	686 ± 198	655 ± 180	610 ± 175	<0.001
Magnesium (mg/day, 1,000 kcal)^b^	174 ± 60	160 ± 61	144 ± 50	124 ± 51	<0.001
Zinc (mg/day, 1,000 kcal)^b^	5.9 ± 2.9	5.5 ± 2.3	5.4 ± 2.3	4.8 ± 2.3	<0.001
Selenium (μg/day, 1,000 kcal)^b^	62.3 ± 28.3	56.0 ± 21.6	53.5 ± 20.2	49.3 ± 17.3	<0.001

*Values are means ± standard deviations*.

a *Dietary protein, carbohydrates and fats are expressed as percentages of total daily energy intake*.

b *For adjusting energy intake, nutrients intake expressed as grams, milligrams, or micrograms per 1,000 kcal*.

c *Analysis of variance was used to test the differences in nutrient intake according to quartile of UPF consumption*.

**Figure 2:**
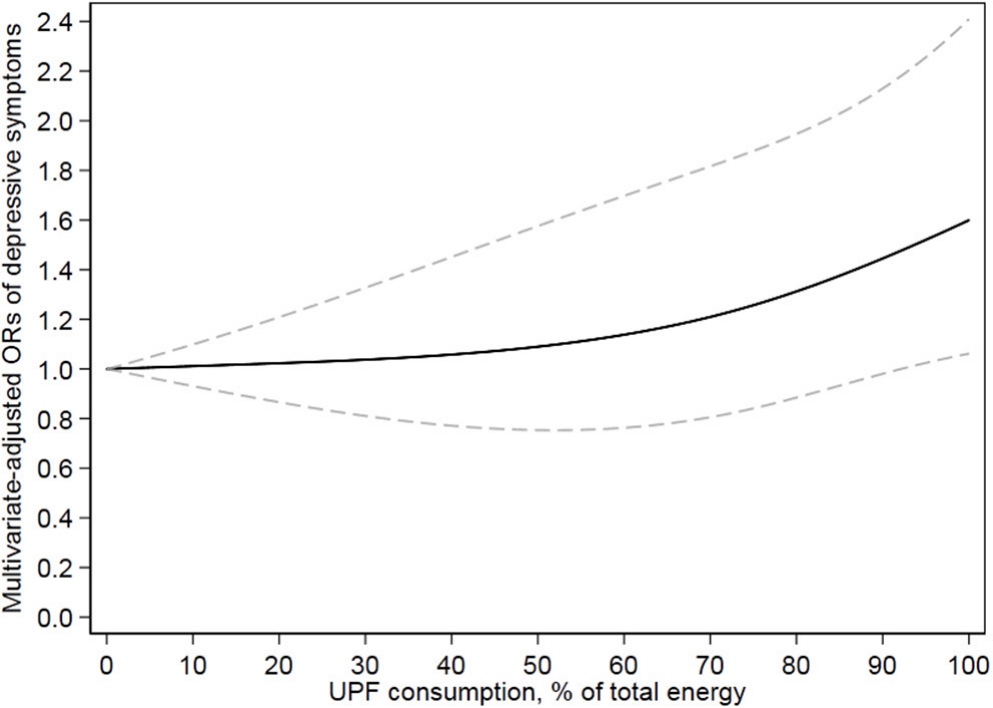
Dose–response relationship of ultra-processed food and risk of depressive symptoms. Model adjusted for age, sex, race, BMI, educational level, annual family income, marital status, physical activity, drinking, smoking, current hypertension, diabetes history, heart disease history, and chronic bronchitis. Solid line and dash line represent the estimated relative risks and their 95% CIs, respectively.

Table 3 presents the association between UPF consumption and depressive symptoms. Without adjusting any covariates, a significantly positive association (*p* = 0.002) was observed between UPF consumption and depressive symptoms; the crude OR with 95% CI was 1.43 (1.10–1.85) for the highest vs. lowest quartile. Model 1 adjusted age and sex, showing the same results as the unadjusted model. Further adjusting for BMI, race, marital status, educational level, family income, smoking, drinking, hypertension, diabetes, heart disease, and chronic bronchitis, the results were still stable; the OR (95% CI) of UPF consumption and depressive symptoms was 1.34 (1.00–1.78) for the highest vs. lowest quartile in the fully adjusted model. Dose–response relationship between UPF consumption and depressive symptoms is shown in Figure 2. In the restricted cubic spline model, a positively linear association was found between the two (*p* for non-linearity = 0.34). Stratified analyses were performed to assess whether this association was modified by physical activities in Table 4. In models 2 and 3, this positive association between UPF consumption and depressive symptoms was only significant in people with poor physical activity. Among physically active people, the effect on depressive symptoms of UPF was small and not significant.

**Table 3:** Weighted odds ratios (95% confidence intervals) for depressive symptoms across quartiles of UPF% (*N* = 13,637).

	**Cases/participants**	**UPF% range**	**Crude**	**Model 1**	**Model 2**
**UPF (%)**					
Quartile 1	272/3,392	<37%	Ref	Ref	Ref
Quartile 2	299/3,532	37–<55%	1.00 (0.76–1.30)	1.00 (0.76–1.31)	1.07 (0.79–1.44)
Quartile 3	275/3,317	55–<73%	1.16 (0.89–1.52)	1.17 (0.90–1.53)	1.16 (0.87–1.54)
Quartile 4	362/3,396	≥73%	1.43 (1.10–1.85)^**^	1.43 (1.10–1.85)^**^	1.34 (1.00–1.78)^*^
*P*^c^			0.002	0.003	0.03

*Model 1 adjusted for age and sex*.

*Model 2 adjusted for age, sex, race, BMI, educational level, annual family income, marital status, physical activity, drinking, smoking, current hypertension, diabetes history, heart disease history, and chronic bronchitis*.

c *P for linearity was calculated by using the median value of each quartiles as a continuous variable in each model*.

* *p < 0.05*;

** *p < 0.01*.

**Table 4:** Weighted odds ratios (95% confidence intervals) for depressive symptoms according to quartiles of UPF%, stratified by physical activity levels.

	**Cases/participants**	**Crude**	**Model 1**	**Model 2**
**Active**				
Quartile 1	36/602	Ref	Ref	Ref
Quartile 2	26/562	0.38 (0.18–0.83)	0.38 (0.17–0.83)^*^	0.36 (0.16–0.83)^*^
Quartile 3	38/586	0.96 (0.47–1.93)	0.98 (0.48–2.01)	0.85 (0.41–1.76)
Quartile 4	48/585	0.89 (0.51–1.67)	0.87 (0.49–1.57)	0.76 (0.39–1.45)
*P*^c^		0.77	0.63	0.91
**Inactive**				
Quartile 1	219/2,395	Ref	Ref	Ref
Quartile 2	218/2,369	0.97 (0.71–1.34)	0.97 (0.70–1.33)	1.07 (0.76–1.51)
Quartile 3	224/2,327	1.22 (0.92–1.62)	1.19 (0.90–1.59)	1.21 (0.92–1.60)
Quartile 4	280/2,375	1.45 (1.10–1.90)^**^	1.41 (1.07–1.86)^*^	1.36 (1.02–1.82)^*^
*P*^c^		0.002	0.003	0.01
**Moderate active**				
Quartile 1	25/460	Ref	Ref	Ref
Quartile 2	32/466	1.42 (0.62–3.25)	1.51 (0.69–3.32)	1.83 (0.89–3.76)
Quartile 3	28/455	1.10 (0.43–2.80)	1.17 (0.47–2.87)	1.18 (0.48–2.88)
Quartile 4	34/455	1.69 (0.73–3.88)	1.81 (0.84–3.93)	1.83 (0.87–3.87)
*P*^c^		0.34	0.19	0.22

*Model 1 adjusted for age and sex*.

*Model 2 adjusted for age, sex, race, BMI, educational level, annual family income, marital status, physical activity, drinking, smoking, current hypertension, diabetes history, heart disease history, and chronic bronchitis*.

c *P for linearity was calculated by using the median value of each quartile as a continuous variable in each model*.

* *p < 0.05*;

** *p < 0.01*.

Sensitivity analysis only including participants with self-reported “usual intake” showed similar results (Supplementary Table 2). In addition, when both participants with PHQ-9 score ≥ 10 and antidepressant users considered as depressive symptoms (Supplementary Table 3), the positive association between UPF consumption and depressive symptoms was more significant.

## Discussion

In this study, we found UPF consumption was positively associated with depressive symptoms in US adults. After adjusting for sociodemographic characteristics, health behaviors, and chronic disease, participants whose UPF contributed more than 73% of total energy intake had a 35% higher risk of depressive symptoms compared with whose UPF contributed <34% of total energy intake.

Several studies evaluated the effect of UPF or partial components on depressive symptoms. One study from the French NutriNet-Santé cohort reported that high UPF consumption was positively associated with depressive symptoms (27). Another prospective study from the Spain SUN cohort, although conducted in specific university graduates, also found a consistent positive association (26). Nevertheless, both studies were conducted in a population with relatively low UPF consumption; 32% contribute to the total energy in the NutriNet-Santé study, and 276 g/day in the SUN study (vs. 55% and 943 g/day in this study). Our results showed that this positive association still existed in the US population with relatively high UPF consumption. In addition, in Whitehall's study (34), a dietary pattern, mainly containing some typical UPF, for instance, sweetened desserts, fried food, processed meat, refined grains, and high-fat dairy products, was associated with the increased risk for depressive symptoms. In contrast, an association between “processed” pattern and depressive symptoms was non-significant in another UK longitudinal study (35).

This positive association between UPF consumption and depressive symptoms could be explained by the following reasons. First, as a typical part of western dietary pattern, although the nutritional value of different types varies greatly, UPF is often accompanied by low diet quality. A NHANES study reported an inverse dose–response association between UPF and overall diet quality (4). Another study also suggested that reducing the intake of UPF was a potentially effective measure to improve the nutritional quality (8). Low diet quality is widely recognized as a risk factor for depression (36). In this study, as the increase of UPF consumption, the content of nearly all “healthy nutrients,” such as zinc, iron, copper, selenium, dietary fiber and vitamins, also presented the obvious declined trend, many of which are considered to be protective factors of depression (37, 38).

Beyond limited nutritional intakes, high consumption of UPF interfered with the intake of “healthy foods” or minimally processed foods (39), declining the diet quality indirectly. Besides, food additives and neo-formed contaminants derived from processing may also contribute to depressive symptoms. Phthalates and bisphenols are widely used as plasticizers in food packaging; a recent study reported that UPF consumption was associated with higher urinary phthalate metabolites concentrations; some researchers believe that exposure to phthalates would increase the risk of depressive symptoms. Moreover, in a study of Korean teenagers and children, artificial sweetener consumption was related to the increased θ-β ratio (ratios of the θ and β waves in the frontocentral brain areas), which is considered to be linked to some negative emotions, including depressive symptoms (40).

The adverse effect of UPF on the gut microbiome might also contribute to depressive symptoms. As the “virtual endocrine organ” (41), the gut microbiome ferments dietary fiber into short-chain fatty acids that are beneficial to normal intestinal function (42). Poor nutritional quality of UPF may lead to a reduction of probiotics (43). Additionally, some additives could also impact the composition and function of the gut microbiome. An animal experiment found that food-grade titanium dioxide, as a whitening agent, could affect bacterial metabolism and promote biofilm formation to impact bacterial function, although it had little effect on gut microbial composition (44). Impaired gut microbiome may cause intestinal metabolism disorder and inflammatory bowel disease and then affect the central nervous system through the microbiome–gut–brain axis (45, 46), leading to the increased risk of depressive symptoms.

In this study, the association between UPF and depressive symptoms is more significant among inactive people, which may be mediated by obesity. Previous literature has described a positive association between UPF and obesity (25, 47, 48).

This study has several advantages. First, the sample from NHANES is large-size and nationally representative, in favor of reliable results. Additionally, we adjusted for many potential related factors of depressive symptoms in logistic regression models for reducing the interference of covariates as far as possible. However, we have to admit that there are some limitations to this study. Reverse causality is a major limitation of this study; a cross-sectional study was restricted to make causal inferences. Second, food processing methods are many and varied; the degree of processing is difficult to quantify. For some foods, such as canned fruits, it is hard to classify them precisely. Third, the dietary survey in this study was not specially designed to distinguish the degree of food processing; a certain degree of misclassification bias existed inevitably. Fourth, one single 24-h dietary recall may not reflect participants' daily diet precisely (49), and the accuracy of a 24-h dietary recall interview is largely dependent on participants' memory. Poor memory of depressive participants may lead to low dietary intake reporting, making the positive association null. Additionally, the PHQ-9 depression scale is a self-assessment scale reflecting the recent mental state of subjects, not a clinical diagnostic standard for depression. Compared with healthy subjects, people with depressive symptoms may be more reluctant to reply to the scale and cooperate with the research survey, resulting in non-response bias. Indeed, in NHANES, some adults did not respond to the PHQ-9.

In conclusion, a positive association was found between UPF consumption and the risk of depressive symptoms in this study. It is warranted to confirm this cross-sectional association prospectively in the US population. Besides, not only the nutritional quality but also non-nutritional factors may play a role in this positive association. Further studies will be needed to explore specific food additives or neo-formed contaminants' impact on depressive symptoms.

## Data Availability Statement

Publicly available datasets were generated in this study. This data can be found here: https://www.cdc.gov/nchs/nhanes/index.htm.

## Ethics Statement

The studies involving human participants were reviewed and approved by NCHS Research Ethics Review Board. The patients/participants provided their written informed consent to participate in this study.

## Author Contributions

LZ and DZ: conceptualization. LZ and XY: data curation. JS: methodology. LZ: writing—original draft. DZ: writing—review and editing. All authors: contributed to the article and approved the submitted version.

## Conflict of Interest

The authors declare that the research was conducted in the absence of any commercial or financial relationships that could be construed as a potential conflict of interest.

## Abbreviations

UPF: ultra-processed foods
NHANES: National Health and Nutrition Examination Surveys
FNDDS: Food and Nutrient Database for Dietary Studies
PHQ-9: Nine-item Patient Health Questionnaire
BMI: Body mass index
OR: odds ratio
CI: confidence intervals
PUFAs: polyunsaturated fatty acids.
