# Supplementary material for: Ultra-Processed Food Is Positively Associated With Depressive Symptoms Among United States Adults
Source: Front Nutr. 2020 Dec 15;7:600449. doi: 10.3389/fnut.2020.600449 (PMC7770142; doi:10.3389/fnut.2020.600449)
Supplement: Supplementary file 1 [file Table_1.DOCX]

Supplementary Material

**Details of Food Classification**

We classify all foods according to "WWEIA category description", "Main Food Code Description" and “SR Code Description” obtained from the USDA Food and Nutrient Database for Dietary Studies (FNDDS).

What we eat in the United States (WWEIA) food category provides an application to analyze the foods and beverages consumed in the U.S. diet. In WWEIA, all of foods and drinks reported in NHANES were classified into a 150 distinct food category. (1) We linked each 8-digit food code in FNDDS with the corresponding WWEIA category, so as to quickly classify most of food items. Foods that can be clearly identified as UPFs by WWEIA category are selected out first, such as ice cream, sweetened beverages, condiments and sauces. Main Food Code Description and SR Code Description were used when WWEIA category can not distinguish the degree of food processing.

According to NOVA, the following foods were included in UPFs category: Breads, ready-to-eat cereals, bacon, reconstituted meat or fish products, frankfurters, sausages, pizza, sandwiches, crackers, French fries and other fried white potatoes, savory snacks, snack/seal bars, cakes, pies, cookies, brownies, doughnuts, sweet rolls, pastries, candy, ice cream, pudding, canned soups, flavored milk, diet beverages, sweetened beverages, liquor and cocktails, margarine, cream and cream substitutes, condiments and sauces, sugar substitutes, protein and nutritional powders.

Moreover, we took into account “Combination Food Type” and “Source of food”, “Frozen meals” or “Lunchables”, or foods acquired from “Restaurant fast food/pizza” or “Vending machine" were also recognized as UPFs.

Two authors independently classified food items and resolved the differences through discussion.

**Reference**

1. U.S. Department of Agriculture ARS. What We Eat in America Food Categories 2011-2012, 2013-2014, 2015-2016 (2015). Available from: <www.ars.usda.gov/nea/bhnrc/fsrg>.

| **Table 1.** Descriptive characteristics of the study participants, NHANES 2011–2016. (N=13637) ^a^ | | | | | | |
| --- | --- | --- | --- | --- | --- | --- |
|  | **All participants** | **Ultra-processed food consumption (% of total energy intake)** | | | |  |
|  |  | **Quartile 1** | **Quartile 2** | **Quartile 3** | **Quartile 4** |  |
|  | N=13637 | N=3392 | N=3532 | N=3317 | N=3396 | ***p* value** |
| **Age (%) ^b^** |  |  |  |  |  | < 0.001 |
| 20~44 years | 5687 (43.0) | 1230 (39.2) | 1345 (42.2) | 1417 (38.6) | 1695 (51.9) |  |
| 45~59 years | 3416 (29.2) | 887 (29.5) | 883 (30.3) | 855 (31.0) | 791 (25.9) |  |
| 60 years and over | 4534 (27.9) | 1275 (31.3) | 1304 (27.4) | 1045 (30.4) | 910 (22.2) |  |
| **Gender (%) ^b^** |  |  |  |  |  | 0.004 |
| Male | 6848 (50.0) | 1756 (49.7) | 1725 (49.5) | 1719 (51.3) | 1648 (49.1) |  |
| Female | 6789 (50.0) | 1636 (50.3) | 1807 (50.5) | 1598 (48.7) | 1748 (50.1) |  |
| **Race (%) ^b^** |  |  |  |  |  | < 0.001 |
| Hispanic | 3287 (14.3) | 934 (16.3) | 928 (15.2) | 746 (12.8) | 679 (13.0) |  |
| Non-Hispanic White | 3425 (66.6) | 1125 (61.5) | 1404 (67.6) | 1436 (69.0) | 1460 (67.6) |  |
| Non-Hispanic Black | 3063 (11.0) | 626 (9.6) | 730 (9.6) | 726 (10.6) | 981 (14.2) |  |
| Non-Hispanic Asian | 1414 (5.1) | 601 (9.6) | 357 (4.6) | 294 (4.1) | 162 (2.5) |  |
| Other races | 448 (3.0) | 106 (3.0) | 113 (3.0) | 115 (3.5) | 114 (2.7) |  |
| **BMI (%) ^b^** |  |  |  |  |  | < 0.001 |
| < 25 kg/m^2^ | 3867 (29.1) | 1079 (31.9) | 1038 (30.0) | 895 (28.3) | 855 (26.3) |  |
| 25 to < 30 kg/m^2^ | 4372 (33.1) | 1152 (35.4) | 1153 (33.7) | 1077 (33.6) | 990 (29.8) |  |
| ≥ 30 kg/m^2^ | 5281 (37.8) | 1143 (32.7) | 1306 (36.3) | 1314 (38.1) | 1518 (43.9) |  |
| **Marital status (%) ^b^** |  |  |  |  |  | < 0.001 |
| Married/Living with partner | 7900 (61.3) | 2040 (62.9) | 2099 (64.3) | 1966 (61.6) | 1795 (56.2) |  |
| Widowed//Divorced/Separated/  Never married | 5732 (38.7) | 1350 (37.1) | 1431 (35.7) | 1351 (38.4) | 1600 (43.8) |  |
| **Educational level (%) ^b^** |  |  |  |  |  | < 0.001 |
| < high school | 2937 (14.3) | 811 (15.3) | 790 (14.6) | 638 (12.5) | 698 (15.0) |  |
| high school | 3013 (21.3) | 676 (19.7) | 737 (20.5) | 1. (1.3) | 872 (3.5) |  |
| < high school | 7684 (64.4) | 1903 (65.0) | 2005 (64.9) | 1950 (66.2) | 1826 (61.5) |  |
| **Annual family income (%) ^b^** |  |  |  |  |  | 0.001 |
| < $20000 | 3236 (18.4) | 821 (18.6) | 792 (17.5) | 768 (17.2) | 855 (20.6) |  |
| $20000 to < $45000 | 4141 (27.8) | 989 (27.1) | 1047 (26.3) | 1019 (28.3) | 1086 (29.6) |  |
| $45000 to < $75000 | 2388 (20.5) | 606 (20.3) | 598 (20.2) | 583 (20.3) | 601 (21.0) |  |
| ≥ $75000 | 3263 (33.3) | 798 (34.0) | 921 (36.0) | 820 (34.2) | 724 (28.8) |  |
| **Physical activity (%) ^b^** |  |  |  |  |  | < 0.001 |
| Active | 2335 (17.0) | 517 (17.2) | 580 (17.1) | 584 (17.3) | 654 (20.1) |  |
| Moderate active | 1836 (15.1) | 479 (16.4) | 447 (14.7) | 456 (15.6) | 454 (14.1) |  |
| Inactive | 9466 (66.9) | 2396 (66.4) | 2505 (68.1) | 2277 (67.1) | 2288 (65.9) |  |
| **Smoking status (%) ^b^** |  |  |  |  |  | < 0.001 |
| Current smoker | 2732 (19.6) | 588 (18.2) | 644 (18.6) | 674 (19.2) | 826 (22.2) |  |
| Former smoker | 3257 (25.3) | 863 (27.7) | 899 (27.1) | 815 (25.0) | 680 (21.4) |  |
| Never smoker | 7636 (55.1) | 1939 (54.1) | 1985 (54.3) | 1826 (55.8) | 1886 (56.4) |  |
| **Had at least 12 alcohol drinks a year (%) ^b^** | 9829 (78.1) | 2406 (78.3) | 2525 (79.2) | 2449 (78.8) | 2449 (76.2) | 0.166 |
| **Current hypertension (%) ^b^** | 5233 (36.1) | 1364 (38.4) | 1378 (37.2) | 1276 (35.6) | 1215 (33.1) | 0.001 |
| **Ever had diabetes (%) ^b^** | 1878 (10.5) | 468 (9.7) | 509 (11.2) | 461 (11.3) | 440 (9.9) | 0.598 |
| **Ever had** **heart disease (%) ^b^** | 1138 (7.0) | 284 (7.1) | 323 (8.1) | 254 (6.6) | 277 (6.3) | < 0.001 |
| **Ever had chronic bronchitis (%) ^b^** | 781 (6.1) | 164 (5.7) | 203 (5.8) | 177 (5.8) | 237 (6.9) | 0.009 |
| ^a^ All percentages and means are weighted to be nationally representative.  ^b^ Categorical variables are represented as number of subjects (weighted percentage).  ^c^ Continuous variables are represented as means (standard errors). | | | | | | |

| **Table S2.** Sensitivity Analysis I: Weighted odds ratios (95% confidence intervals) for depressive symptoms across quartiles of UPF% (Only participants who reported "usual intake" in the dietary survey were included, n=9661). | | | | | | |  |  |
| --- | --- | --- | --- | --- | --- | --- | --- | --- |
|  | **Cases/Participants** | | **Crude** | **Model 1** | **Model 2** | | | |
| **UPF (%)** |  | |  |  |  | | |  |
| Quartile 1 | 156/2509 | | Ref | Ref | Ref | | |  |
| Quartile 2 | 182/2583 | | 1.06 (0.78-1.43) | 1.08 (0.79-1.46) | 1.16 (0.81-1.65) | | |  |
| Quartile 3 | 164/2382 | | 1.20 (0.91-1.9) * | 1.21 (0.92-1.59) | 1.19 (0.88-1.61) | | |  |
| Quartile 4 | 205/2187 | | 1.58 (1.13-2.20) ** | 1.59 (1.13-2.25) ** | 1.50 (1.02-2.20) * | | |  |
| *P*^c^ |  |  | < 0.001 | 0.008 | 0.04 | | |  |
| Model 1 adjusted for age and gender.  Model 2 adjusted for age, gender, race, BMI, educational level, annual family income, marital status, physical activity, drinking, smoking, current hypertension, diabetes history, heart disease history and chronic bronchitis.  ^c^ P for linearity was calculated by using the median value of each quartile as a continuous variable in each model.  * p < 0.05; ** p < 0.01. | | | | | |  |  |  |

| **Table S3.** Sensitivity Analysis II: Weighted odds ratios (95% confidence intervals) for depressive symptoms across quartiles of UPF% (Both participants with PHQ-9 score ≥10 or participants using antidepressants are considered as depressive symptoms) | | | | | | |  |  |
| --- | --- | --- | --- | --- | --- | --- | --- | --- |
|  | **Cases/Participants** | | **Crude** | **Model 1** | **Model 2** | | | |
| **UPF (%)** |  | |  |  |  | | |  |
| Quartile 1 | 272/3392 | | Ref | Ref | Ref | | |  |
| Quartile 2 | 299/3532 | | 1.04 (0.85-1.27) | 1.05 (0.86-1.28) | 1.02 (0.83-1.26) | | |  |
| Quartile 3 | 275/3317 | | 1.24 (1.00-1.53) * | 1.28 (1.04-1.59) * | 1.22 (0.99-1.50) | | |  |
| Quartile 4 | 362/3396 | | 1.51 (1.21-1.89) ** | 1.63 (1.30-2.04) ** | 1.51 (1.18-1.92) ** | | |  |
| *P*^c^ |  |  | < 0.001 | < 0.001 | < 0.001 | | |  |
| Model 1 adjusted for age and gender.  Model 2 adjusted for age, gender, race, BMI, educational level, annual family income, marital status, physical activity, drinking, smoking, current hypertension, diabetes history, heart disease history and chronic bronchitis.  ^c^ P for linearity was calculated by using the median value of each quartile as a continuous variable in each model.  * p < 0.05; ** p < 0.01. | | | | | |  |  |  |

| **Table S4.** Sensitivity Analysis III: Weighted odds ratios (95% confidence intervals) for depressive symptoms across quartiles of UPF%. (UPF consumption is presented in gram per day) | | | | | | | | | | |  |  |
| --- | --- | --- | --- | --- | --- | --- | --- | --- | --- | --- | --- | --- |
|  | **Cases/Participants** | | **UPF range** | | **Crude** | | **Model 1** | | **Model 2** | | | |
| **UPF (g/day)** |  |  | |  | |  | |  | | | |  |
| Quartile 1 | 290/3410 | <394 | | Ref | | Ref | | Ref | | | |  |
| Quartile 2 | 271/3409 | 394 to <742 | | 0.99 (0.73-1.34) | | 1.00 (0.74-1.36) | | 1.05 (0.74-1.49) | | | |  |
| Quartile 3 | 301/3409 | 742 to <1230 | | 1.08 (0.82-1.43) | | 1.13 (0.85-1.50) | | 1.19 (0.87-1.64) | | | |  |
| Quartile 4 | 346/3409 | ≥1230 | | 1.33 (1.07-1.66) ** | | 1.48 (1.18-1.87) ** | | 1.39 (1.06-1.83) * | | | |  |
| *P*^c^ |  |  | | 0.001 | | < 0.001 | | 0.004 | | | |  |
| Model 1 adjusted for age and gender.  Model 2 adjusted for age, gender, race, BMI, educational level, annual family income, marital status, physical activity, drinking, smoking, current hypertension, diabetes history, heart disease history and chronic bronchitis.  ^c^ P for linearity was calculated by using the median value of each quartile as a continuous variable in each model.  * p < 0.05; ** p < 0.01. | | | | | | | | | |  |  |  |
